# High Potencies in Parturition

**Published:** 1881-11

**Authors:** Julius Schmitt

**Affiliations:** Rochester, N. Y.


### CLINICAL BUREAU.

### HIGH POTENCIES IN PARTURITION.

JULIUS SCHMITT, M. D., ROCHESTER, N. Y.

CASE 1. Mrs. A., aged 25, blonde, robust, leuco-phlegmatic temperament; pains commenced in the afternoon of the 12th of September, 1878. I saw her first at 11 P. M., and found her in the following condition: Pains, very feeble and irregular, os uteri very little dilated, bag of waters commenced to form; head presentation. She was in a whining and complaining mood. A dose of *Pulsatilla* (3) was given, and in twenty minutes the pains became regular and her mental condition grew normal and more quiet. Labor progressed for an hour, os uteri dilated naturally, and the bag of waters protruded powerfully during every pain. I thought another dose would do her still more good (this being the first labor-case which I attended after investigating Homœopathy) and accordingly another dose of *Pulsatilla* (3) was administered. Within a quarter of an hour the pains became really terrific, the head became at once well wedged in the superior straight; the os dilated rapidly, the bag of waters ruptured; one pain lasting 1½--2 minutes (it seemed to me an eternity), drove the head down into the pelvis, extended the face into the hollow of the sacrum, pushed the occiput under the symphysis pubis, swept the face over the soft parts of the pelvis, and did not cease until the whole child was born. I told the patient to stop her bearing down, but "I can't," she gasped, and on went this bearing of a child, as I had never seen before in my life. The placenta came away naturally in a short time afterwards, and the patient made a good recovery.

On the 7th of November, 1880, this same lady came into labor again. Pains set in in the evening, and I saw her at 2 A. M., the next morning. She was in the same condition as when I saw her with her last child. Pains irregular, and causing her to whine and complain. A dose of *Pulsatilla* 2 C changed the scene in ten minutes. She grew jovial, her natural condition, and the pains became regular; os uteri dilated almost as far as it naturally can, when again the pains grew feeble and irregular; another dose of *Pulsatilla* 2 C did not improve them, but brought out the following symptoms, viz.: Pains would appear *very suddenly, last a short time and then cease, just as suddenly as they came,* her face became almost purple with the pains, and eyes congested. The os uteri, which had been soft and yielding heretofore, was found to exhibit a very sharp, unyielding edge and felt very hot to the examining finger. The patient received a dose of *Belladonna* CM (Swan); the very next pain showed improvement, in being longer, and not so abrupt in coming and going; the next two pains were still better, and the fourth pain after receiving the dose did not cease until the child was born. Thus *Belladonna* produced exactly the same effect as *Pulsatilla*, two years ago, with this difference: two doses of *Pulsatilla* (3) were required in order to do the same thing as one dose of *Belladonna* CM.

The patient made a good recovery without any more medicine, except a dose of *Arnica* CM, which was called for by the soreness of the abdominal muscles.

Case 2. On the 16th of November, 1879, Mrs. W., aged 36, robust, leuco-phlegmatic temperament, expected her sixth child. I had attended her in two previous confinements, which had been very tedious, on account of my ignorance of Nature's law of cure. I saw her at 3.30 P. M. She was up and around, complaining of very slight pains, which, however, were accompanied by *urging to stool and to micturition.* The examining finger detected the os uteri high up, dilated to the size of a quarter of a dollar, the head presenting, One dose of *Nux vomica* CM (Swan) was given dry on the tongue. The pains commenced to improve within a quarter of an hour, becoming more effective, minus the pressure upon rectum and bladder. At a quarter past 4 o'clock they had grown so strong, that I ordered the patient to bed. In the act of undressing the amniotic fluid burst away, and the pains became violent immediately. On examination I found the head well wedged into the superior straight, os uteri fully dilated, but its anterior lip swollen, and œdematous; forming a rounded cushion behind the symphysis pubis. My former experience in this complication made me think of an incision with a sharp pointed bistoury, but this was not necessary. The expulsive pains pressed violently against this bullwark of obstruction, and in a few minutes a powerful pain burst the swelling, and did not stop until the child was delivered. This occurred at 4.30 o'clock, P. M., just an hour after the dose of *Nux vomica* had been given. The placenta was delivered spontaneously, five minutes later. The patient made a good recovery without further medication, except the usual dose of *Arnica*.

CASE 3. Mrs. C., primipara, dark complexion, excitable temperament, commenced her first labor on the 29th of April, 1881, and was attended by a German midwife, who sent for me on the morning of the 30th of April. I saw the patient at 8 A. M., and found very irregular pains, os only partially dilated and unyielding; bag of waters had ruptured at 3 A. M., head presented at the superior straight. While having a pain, the patient kept up a continuous cry: "*Oh my back, my back, help me, oh my back!*" Here was an unmistakable indication for *Causticum*, as given by our great Guernsey and this remedy was administered in a single dose of the CM (Swan) potency, dry on the tongue. I left her with the midwife, telling them that I should return in about an hour, but that I thought by that time the child would be born. At 10 o'clock I entered the room again and came just in time to witness the last strong pain, which expelled the child. The midwife told me that the pains changed soon after my departue. This patient also recovered without further medication.

CASE 4. On the 3d of June, 1881, Mrs. E., dark complexion and even temperament, in labor with her fifth child, required my assistance, the midwife in attendance not being able to do anything more for her. Having known the patient for a good many years, I believed her to be one of those women who are brave and plucky and try to get through difficulties as quietly as possible; in fact, while driving there I thought she would possibly require *Lycopodium*. How astonished was I, therefore, to find her *perfectly demoralized* and *crying for help*, the pains being irregular and spasmodic and *entirely unbearable to her*. The vagina was very hot and so was the os uteri, the latter partially dilated and dilatable, head of child ready to be pushed down, only lacking the appropriate *vis a tergo*. The patient's pulse was 120 and full, her skin was hot and dry, mouth dry and parched. Desire for cold water. I put a dose of *Chamomilla* CM (Swan) on her tongue, and knowing that she always reacted quickly to the *simillimum*, kept my watch in hand to observe the effect. In two minutes she fell into a little doze, which lasted one minute, then there followed three good pains in the next two minutes, the third pain delivering the child, which was small, weighing hardly six pounds. Placenta came away naturally and she did very well until the 11th of June, when I was again summoned. The following condition presented itself: Dull pain in right loin, going around to front, becoming sharp, as if a stick was pushed through from behind forwards, if touched. *Better from keeping perfectly quiet;* a cough, short and dry, increases the pain and produces also a fine stitch in right hip. Cough *worse from speaking, better from keeping quiet.* Backache, when lying on either side, but also worse when lying long on the back. *Cannot stay in one position for any length of time;* headache as if from a tight cap on head, goes across forehead to each temple, *scalp very sore to touch.*

*Thirst very great, and drinks much at a time. Urine dark brown and turbid.* Pulse, 76. *Tongue coated white. Bryonia* CM (Swan), one dose right away, a second one twelve hours later. On the 13th of June felt better in every respect, was out of bed and got well without further medicine.

